# Estimating sodium and potassium intakes in a Portuguese adult population: can first-morning void urine replace 24-hour urine samples?

**DOI:** 10.1017/jns.2025.16

**Published:** 2025-03-26

**Authors:** Ana Carolina Lages Goios, Milton Severo, Carla Maria Moura Lopes, Duarte Paulo Martins Torres

**Affiliations:** 1 Faculty of Nutrition and Food Sciences, University of Porto, Rua do Campo Alegre, 823, 4150-180 Porto, Portugal; 2 Epidemiology Research Unit, Institute of Public Health, University of Porto, Rua das Taipas 135, 4050-091 Porto, Portugal; 3 Instituto de Ciências Biomédicas Abel Salazar, Universidade do Porto, Rua Jorge de Viterbo Ferreira 228, 4050-313 Porto, Portugal; 4 Laboratório para a Investigação Integrativa e Translacional em Saúde Populacional (ITR), Universidade do Porto, Rua das Taipas, n◦ 135, 4050-600 Porto, Portugal; 5 Department of Public Health and Forensic Sciences, and Medical Education, Faculty of Medicine, University of Porto, Alameda Prof. Hernâni Monteiro, 4200-319 Porto, Portugal

**Keywords:** Dietary intake, Potassium, Sodium, Spot urine samples, Urine specimen collection, 24-h urine excretion, 24-h, 24-hour, BMI, body mass index, CI, confidence interval, CVD, cardiovascular diseases, D, day, FMV, first-morning void, IAN-AF, National Food, Nutrition and Physical Activity Survey, PABA, para-aminobenzoic acid, SD, standard deviationl, USA, United States of America, WHO, World Health Organization

## Abstract

This study aimed to assess the extent to which first-morning void (FMV) urine samples can estimate sodium and potassium excretion compared with 24-hour (24-h) urine samples at the population level. We conducted a cross-sectional study collecting urine samples (FMV and 24-h) and two non-consecutive 24-h dietary recalls in a sub-sample from the Portuguese IAN-AF sampling frame. Six predictive equations were used to estimate 24-h sodium and potassium excretion from FMV urine samples. Pearson correlation coefficients were calculated to compare the association between FMV and 24-h urine collections. Cross-classifications into tertiles were computed to calculate the agreement between measured and estimated excretion with and without calibration. Pearson correlation coefficients were calculated to compare the excretion estimation from FMV and reported intake from 24-h dietary recalls. Bland–Altman plots assessed the agreement between two-day dietary recall and the best-performing calibrated equation. Data from eighty-six subjects aged 18–84 were analysed. Estimated sodium and potassium concentrations from the predictive equations moderate or strongly correlated with the measured 24-h urine samples. The Toft equation was the most predictive and reliable, displaying a moderate correlation (r=0.655) with no risk of over or underestimation of sodium excretion (p=0.096). Tanaka and Kawasaki equations showed a similar moderate correlation (r=0.54 and r=0.58, respectively) but tended to underestimate the 24-h urine excretion of potassium (p<0.001). Calibrated predictive equations using FMV urine samples provide a moderately accurate alternative and resource-efficient option for large-scale nutritional epidemiology studies when 24-h urine collection is impractical.

## Introduction

High dietary sodium and low dietary potassium intakes are associated with hypertension and increased risk of cardiovascular diseases (CVD)^(1)^. Reducing dietary sodium is estimated to be one of the most effective strategies to improve health and reduce the burden of non-communicable diseases^(2,3)^.

The World Health Organization (WHO) recommends that all adults reduce their sodium intake to <87 mmol (<5 g of salt) per day and increase their potassium intake to ≥90 mmol (≥3.5 g) per day. The WHO World Health Assembly endorsed a 30% reduction of dietary sodium consumption as one of nine major targets to reduce the global burden of non-communicable disease by 25% by 2025^(4)^.

Accurate measuring and monitoring of salt consumption levels enable an objective decision on whether salt reduction needs to be addressed and provides a baseline measure to monitor the effectiveness of salt reduction strategies. However, several international health and scientific organisations have expressed concerns about using inappropriate low-quality research methods to measure sodium intake^(5)^. The International Consortium for Quality Research on Dietary Sodium/Salt was formed to establish recommended minimum standards for research on dietary sodium^(6)^.

Several methods are available to measure dietary sodium, commonly estimated by reports of food consumption or by the analysis of urine samples. Dietary sodium can be estimated using either food frequency questionnaires or a 24-hour (24-h) recall approach, which tends to underestimate sodium intake (recall bias) and rely on accurate information on the sodium content of foods or questionnaires that have been validated for dietary sodium^(7)^. The 24-h urine collection is widely regarded as the ‘gold standard’ method for assessing sodium intake and is often used to compare and validate other methods of sodium intake. Assuming that 24-h urine collections capture between 86% to 93% of average sodium intake, urinary sodium can be reliably used to estimate current 24-h dietary sodium in population studies^(8,9)^. However, the 24-h urine collection is expensive and onerous. Collecting a complete 24-h urine sample is challenging and can compromise its utility in assessing dietary sodium^(10)^. Managing and quality control of population studies must follow rigorous standards to ensure complete urine collection, and no best method of checking completeness is available. Although the use of para-aminobenzoic acid is referred to as the most reliable method for determining completeness^(11)^, the risk of misclassification is still a problem. Collecting 24-h urine has a significant respondent burden, and a sizable proportion of potential respondents may decline to participate in studies that involve 24-h urine collection. The poor response rate can lead to an inaccurate and spurious population estimate of sodium consumption^(12)^ and compromise the feasibility of using 24-h urine collection in large-scale epidemiological studies^(10,13)^.

Spot urine samples have been proposed as a more straightforward potential solution to estimate 24-h urinary sodium excretion and overcome the challenges of collecting 24-h urine samples. Several equations to estimate 24-h urine sodium excretion from spot urine samples have been proposed and used as an alternative to a 24-h urine collection method^(14–20)^. Although numerous studies have highlighted that spot urine samples are inconsistent with urinary sodium excretion measured by 24-h urine collection^(20–23)^, other studies have suggested that spot urine samples may be used to estimate the mean population salt intake with reasonable accuracy^(10,24–26)^.

The established spot urine equations were developed in white, non-Hispanic black and Japanese populations and tested in different populations. The utility of spot urine equations is thus likely to be heterogeneous based on the geographic location, race, and ethnicity of the population studied^(13)^. Several studies have tested these equations on developed countries and Western populations, but there is little evidence of the usefulness of spot urine in the Portuguese adult population. The main objective of our study was to examine whether first-morning void (FMV) samples can provide a reasonable estimate of sodium and potassium intake at the population level, compared to the ‘gold standard’ 24-h urine samples. The secondary objective was to correlate sodium and potassium intake estimation through FMV samples against the one-day and two-day dietary recalls.

## Material and methods

### Study design and participant recruitment

This study comprises a secondary analysis of a sub-sample of data collected from a validation study conducted in Portugal between 2015 and 2016^(27)^.

A total of 95 participants from the same National Food, Nutrition and Physical Activity Survey (IAN-AF) sampling frame were recruited for this study. The methods of the IAN-AF sampling frame are described in detail in previous publications^(28,29)^. Between May and December 2016, healthy men and women aged 18-84 from the same IAN-AF sampling frame were invited to participate in a more detailed validation study by telephone or during the first face-to-face interview. Participant enrolment was assessed using specific eligibility criteria described in a previous study^(27)^. Briefly, the exclusion criteria included: taking diuretics, being pregnant or lactating, having diabetes or kidney disease, haemophilia or any condition requiring supplemental O_2_, donating blood or plasma during or <4 weeks before the study, following prescribed dietary therapy and/ or having had a urinary tract infection within one month or commencing the survey.

A total of 190 urinary samples (24-h urine and paired FVM samples) from 95 participants were collected. As previously described^(27)^, the completeness of the 24-h urine was assessed using two criteria: the 24-h urinary creatinine excretion within the recommended ranges of 14.4-33.6 mg/kg body weight for men and 10.8-25.2 mg/kg body weight for women^(30)^, and a total 24-h urine volume (≥500 mL)^(31)^. Only data from complete collections meeting both criteria were included in the analysis^(32,33)^. Nine participants were excluded according to these coefficient creatinine-based and total urine volume criteria, totalling 86 paired complete samples that were included for analysis.

### Data collection

#### Dietary assessment

Dietary intake was obtained by two non-consecutive 24-h dietary recalls, separated by 8 to 15 days. Structured interviews were performed by trained nutritionists according to the procedure based on the automated multiple-pass method for 24-h dietary recall^(34)^, as described elsewhere^(29)^.

The data were obtained using the previously validated eAT24 software^(27)^, an electronic dietary assessment tool based on a client-server architecture developed for the IAN-AF. This allowed the collection and description of food consumption data by 24-h recalls. All foods, including beverages and dietary supplements, were recorded per eating occasion and quantified and described as eaten. This description required the utilisation of several facets and respective descriptors through the FoodEx2 classification system^(35)^. The place and time of meal consumption were also recorded for each eating occasion.

The software allowed subsequent conversion of foods into nutrients, using the Portuguese food composition table^(36)^ by default, which was continuously adapted and updated. A recipe module was also created, in which the recipes were disaggregated into raw ingredients, allowing the description and quantification of each item. The software was also able to include new food items or new recipes during the data collection process.

For quantification, different methods were available: (i) weight or volume, (ii) standard units, (iii) photographs (food picture book including a 186 food photograph series (with six portions/food per recipe) and a household measures photograph series^(37)^, (iv) household measures and (v) default portions.

The participants received detailed written and oral instructions and the necessary equipment for collecting urine samples. Anthropometric data (body weight and height) were collected using standardised procedures^(29)^. Participants were instructed to bring their urine samples to the second interview when a second 24-h dietary recall was applied.

#### Urine collection and processing

We asked participants to collect urine samples on the day preceding the second interview. Urine samples were collected in two separate containers. The first container (2700 mL, identified as container A) contained all urine passed the day before the interview, except the first void of that morning. A second container (500 mL, identified as container B) collected only the first void urine of the day of the second interview (urine sample identified as ‘first-morning void’). No preservatives were added to the urine containers, and participants were instructed to keep the samples refrigerated (4°C) throughout the collection period. During the day of urine collection, all participants also replied to a paper questionnaire that included information on the time elapsed from the beginning and the end of the collection, details of any medication, and whether they had any problems or missed urine collection.

Urine samples were weighed and mixed at the laboratory. The weight of urine from containers A and B was quantified separately, and a proportionally pooled 24-h urine sample (identified as ‘24-h urine’) was prepared by combining samples A and B. From each participant, both urine samples (‘first-morning void’ and ‘24-h urine’) were aliquoted: 1 × 45 mL (in 50 mL Falcon pre-labelled tube) + 10 × 1.5 mL (in 2 mL pre-labelled microtubes). These aliquots were refrigerated immediately before being moved to a –80°C storage within 24 hours for further analysis.

#### Chemical analysis

Sodium and potassium excretions were assessed using an ion-selective electrode Na+ and K+ assay (Beckman Coulter, USA). Further adjustments were made to reflect extra-renal losses of sodium and potassium, estimated at 0.86 and 0.80, respectively^(8)^.

The 24-h urine volume was adjusted for self-reported collection time according to the following expression: 24-h urine volume (mL) = total volume collected (mL)/self-reported collection time (h) × 24^(31)^. Urine density was assumed to be approximately equal to 1.0 g/mL. Urinary creatinine was measured using the Jaffe method (Beckman Coulter, USA).

#### Estimation of sodium (and salt) and potassium intake from 24-h urine and FMV urine samples

Sodium and potassium excretion was estimated from FMV urine samples using a series of established estimation equations: Kawasaki^(14)^, Tanaka^(15)^, Mage^(38)^, Toft^(18)^ and the International Cooperative Study on Salt, Other Factors, and Blood Pressure (INTERSALT) with or without potassium^(16)^ (Supplementary material Table S2). These equations use the concentration of sodium and creatinine in the FMV urine sample, age, weight, and height (or body mass index [BMI]). The Kawasaki, Mage, Toft and INTERSALT methods also have a separate equation for each sex, and Mage includes a correction term for the African-American race. Except for the INTERSALT equations, all other equations are based on the ratio of sodium to creatinine in the FMV urine sample and include an estimate of 24-h creatinine excretion to extrapolate the FMV sodium concentration to a 24-h urine value. The INTERSALT equations use FMV sodium and FMV creatinine concentration, age, BMI, and sex, with or without FMV potassium concentration, to derive 24-h salt intake^(39)^.

To estimate Na and K intakes, the 24-h excretion estimates through predictive equations were divided by 0.86 and 0.80, respectively, to reflect extra-renal losses^(8)^.

### Statistical analysis

Continuous variables are described using mean and standard deviation (SD). Categorical variables are presented as count and relative frequency. Paired samples t-tests were used to compare the mean sodium and potassium excretion measured from 24-h urine with predicted excretion using the equation-based methods applied to sodium and potassium values measured in FMV urine samples. Pearson’s correlation was computed to analyse the association between the estimated 24-h urine excretion of sodium and potassium through predictive equations of FMV urine samples and the measured 24-h excretion of sodium and potassium concentration. Pearson’s correlation was also used to assess the association between estimated sodium and potassium intake from 24-h dietary recalls (one day and the average of two days) and the sodium and potassium concentration estimated using calibrated predictive equations from FMV urine samples. The Pearson correlation coefficients were interpreted as negligible (0.00-0.09), weak (0.10-0.39), moderate (0.40-0.69), strong (0.70-0.89), and very strong (0.90-1.00)^(40)^. The cut-off for all the agreement studies was defined using the tertiles from the 24-h urine measurements (for sodium: 2685, 3775 mg/d and potassium: 2053, 2985 mg/d). Accuracy was defined when the participants were classified in the same category using the 24-h urine measurements and the predictive equations. Cross-classification was performed for each predictive equation to compare the predictive equations with and without calibration and the one-day and the mean of two-days of dietary recalls. The linear weighted Kappa coefficient with the 95% confidence interval (CI) was computed to assess the strength of agreement, considering poor agreement: *K* < 0, slight agreement: 0 <= *K* < 0.2, fair agreement: 0.2 <= *K* < 0.4, moderate agreement: 0.4 <= *K* < 0.6, substantial agreement: 0.6 <= *K* < 0.8, almost perfect agreement: 0.8 <= *K* <= 1^(41)^.

If a bias was identified between the mean value of the 24-h urine and using the predicted equations, a recalibration model was applied to adjust the intercept and the slope of the predicted equations to the Portuguese sample values. 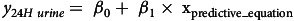 , where y is 24-h sodium or potassium concentration, and x is sodium or potassium concentration estimated from the predictive equation, respectively. The regression coefficients, 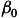 and 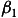 were calculated using linear regression.

The Bland – Altman plots were used to determine the agreement of sodium and potassium estimation between dietary intake from two-day recall and the estimated by the predictive equations from FMV urine. Log transformations to sodium and potassium data were applied before analysis to reduce the skewness of distributions. The difference in sodium and potassium estimation from the two methods is presented on the vertical axis against the mean of the two methods on the horizontal axis. Only the best-performing equation (with the highest correlation coefficient) for sodium and potassium was used to illustrate the difference between these two methods against the mean of the two methods [(intake + excretion)/2].

The significant level was fixed at 0.05. The R 4.2.1 software was used for all statistical analyses.

## Results

Half of the participants (50%) were women, and 12% were aged 65 years or more. Two-thirds of the population was overweight or obese (72% of men and 61% of women). Demographic and anthropometric characteristics and the average energy intake of the participants are presented in supplementary material Table S1.

### Prediction of 24-h urine sodium and potassium from FMV urine samples

The mean and SD of sodium, potassium, and creatine concentration for both FMV and 24-h urine samples are presented in Table 1. The mean total volume of 24-h urine samples was 1406 ± 614 mL.

**Table 1.** First-morning void and 24-h urine samples concentration of sodium, potassium, and creatinine for all participants

	First-morning void urine samples		24-h urine samples	
	Mean	SD	Mean	SD
Sodium, *mmol/L*	118.9	47.6	115.5	44.3
Potassium, *mmol/L*	41.8	19.0	55.1	23.6
Creatinine, *mmol/L*	12.7	6.6	10.7	5.3

SD, Standard Deviation.

The estimated 24-h urine excretion values of sodium and potassium from predictive equations using FMV urine samples are presented in Table 2. The estimation of sodium and potassium excretion from predictive equations was moderate to strongly correlated with measured 24-h urine samples (Table 2). INTERSALT (with or without K) equations showed the strongest correlation with the 24-h urine excretion of sodium (r = 0.70 and r = 0.71, respectively). However, both the INTERSALT (with or without K) and Mage equations tended to underestimate the 24-h urine excretion of sodium (p<0.05). Conversely, the Kawasaki equation overestimated the 24-h urine excretion of sodium (p<0.001). For 24-h urine potassium excretion, both the Tanaka and Kawasaki predictive equations showed a similar moderate correlation (r=0.54 and r=0.58, respectively). Both equations tended to underestimate the 24-h urine excretion of potassium (p<0.001).

**Table 2.** Estimated 24-h urine excretion of sodium and potassium through predictive equations (from first-morning void urine samples) and correlation to measured 24-h excretion

	Mean^c^	SD	p^a^	r (95% CI)^b^
Sodium				
Measured 24-h urine excretion, mg/d	3442.2	1513.9	Ref.	
Estimated 24-h urine excretion through predictive equations				
Tanaka, mg/d	3419.1	794.1	0.864	0.563 (0.399 to 0.693)
Kawasaki, mg/d	4279.5	1207.0	<0.001	0.610 (0.458 to 0.728)
Intersalt (with K), mg/d	3188.0	799.0	0.036	0.703 (0.577 to 0.796)
Intersalt (without K), mg/d	3150.3	796.3	0.016	0.708 (0.583 to 0.800)
Mage, mg/d	2914.3	1813.5	0.003	0.542 (0.373 to 0.676)
Toft, mg/d	3652.5	820.9	0.096	0.655 (0.515 to 0.761)
Potassium				
Measured 24-h urine excretion, mg/d	2726.1	1062.7	Ref.	
Estimated 24-h urine excretion through predictive equations				
Tanaka, mg/d	1527.9	347.2	<0.001	0.545 (0.376 to 0.678)
Kawasaki, mg/d	1875.3	477.6	<0.001	0.584 (0.425 to 0.708)

SD, Standard deviation; 95% CI, 95% confidence interval.

a Paired samples t-tests.

b Pearson’s correlation (r).

c Na concentration was converted to Na excretion according to the following expression: Na intake (mg/d) = (Na concentration in urine (mmol/L) × 24-h urine volume (L) × 23); K concentration was converted to K excretion according to the following expression: K intake (mg/d) = (K concentration in urine (mmol/L) × 24-h urine volume (L) × 39).

The cross-classification results showed a similar trend when comparing the estimated 24-h urine excretion of sodium and potassium from predictive equations of FMV urine to the measured 24-h urine samples (Table 3). However, the agreement (k coefficient) increased when using calibrated prediction equations, except when using the Mage equation for sodium estimation.

**Table 3.** Cross-classifications into tertiles for agreement between measured and estimated 24-h excretion of sodium and potassium through predictive equations from first-morning void urine samples

	Without calibration			With calibration		
	Agreement (%) (95% CI)	*k* (95% CI)	p	Agreement (%) (95% CI)	*k* (95% CI)	p
Sodium						
Tanaka	50.0 (39.7 to 60.3)	0.336 (0.180 to 0.492)	<0.001	51.2 (40.2 to 62.0)	0.353 (0.197 to 0.509)	<0.001
Kawasaki	43.0 (32.5 to 54.1)	0.239 (0.128 to 0.350)	<0.001	55.8 (44.7 to 66.4)	0.429 (0.277 to 0.580)	<0.001
Intersalt (with K)	57.0 (45.9 to 67.5)	0.447 (0.299 to 0.594)	<0.001	57.0 (45.9 to 67.5)	0.486 (0.346 to 0.626)	<0.001
Intersalt (without K)	59.3 (48.2 to 69.6)	0.495 (0.355 to 0.636)	<0.001	58.1 (47.0 to 68.5)	0.497 (0.358 to 0.637)	<0.001
Mage	47.7 (36.9 to 58.7)	0.311 (0.159 to 0.463)	<0.001	43.0 (32.5 to 54.1)	0.222 (0.074 to 0.369)	0.003
Toft	43.0 (32.5 to 54.1)	0.261 (0.136 to 0.386)	<0.001	53.5 (42.5 to 64.2)	0.413 (0.263 to 0.563)	<0.001
Potassium						
Tanaka	34.9 (25.1 to 46.0)	0.032 (-0.016 to 0.081)	0.190	46.5 (45.8 to 57.5)	0.296 (0.147 to 0.445)	<0.001
Kawasaki	44.2 (33.6 to 55.3)	0.213 (0.107 to 0.319)	<0.001	48.8 (38.0 to 59.8)	0.336 (0.186 to 0.486)	<0.001

95% CI: 95% confidence interval; k: Kappa coefficient.

### Correlation of sodium and potassium estimated from FMV urine samples and one-day and two-day dietary recalls

The estimated sodium and potassium intakes from dietary recalls were reported in our previous study^(27)^. The estimated 24-h urine excretion of sodium and potassium from predictive equations of FMV urine samples were poorly correlated with one-day or two-day dietary recalls, showing negligible to moderate correlation (Table 4). The Toft prediction equation was the most effective in estimating the sodium excretion when compared to the one-day or two-day recalls, with a moderate correlation (r=0.411 and r=0.449, respectively), followed by the INTERSALT (with K) with weak and moderate correlation for one-day or two-day recalls (r=0.379 and r=0.436, respectively) and INTERSALT (without K) with weak and moderate correlation for one-day or two-day recalls (r=0.374 and r=0.426, respectively). Other predictive equations provided negligible correlations for estimating sodium excretion. To assess the potassium, the Kawasaki equation showed the best correlation with one-day or two-day dietary recalls but with only a weak correlation (0.315 and 0.347, respectively). Generally, the estimation of sodium and potassium was more correlated with the two-day average than with the one-day recall.

**Table 4.** Correlation between estimated sodium and potassium intake from 24-h dietary recalls (one day of recall and the mean of the first and second day of recall) and estimated through calibrated predictive equations from first-morning void urine samples

	One-day of recall		Mean of two days of recall	
	r (95% CI)^a^	p-value	r (95% CI)^a^	p-value
Sodium				
Tanaka	0.104 (−0.110 to 0.309)	0.340	0.121 (−0.094 to 0.324)	0.269
Kawasaki	0.174 (−0.039 to 0.372)	0.109	0.188 (−0.025 to 0.385)	0.083
Intersalt (with K)	0.379 (0.182 to 0.547)	<0.001	0.436 (0.247 to 0.593)	<0.001
Intersalt (without K)	0.374 (0.176 to 0.543)	<0.001	0.426 (0.235 to 0.585)	<0.001
Mage	0.081 (−0.133 to 0.288)	0.457	0.114 (−0.101 to 0.318)	0.297
Toft	0.411 (0.218 to 0.573)	<0.001	**0.449 (0.262 to 0.603)**	**<0.001**
Potassium				
Tanaka	0.238 (0.027 to 0.428)	0.028	0.259 (0.050 to 0.446)	0.016
Kawasaki	0.315 (0.110 to 0.494)	0.003	**0.347 (0.145 to 0.520)**	**0.001**

95% CI, 95% confidence interval; Boldface indicates the equations with the highest correlation coefficients, representing the most effective estimations for Na or K.

a Pearson’s correlation.

**Figure 1.**
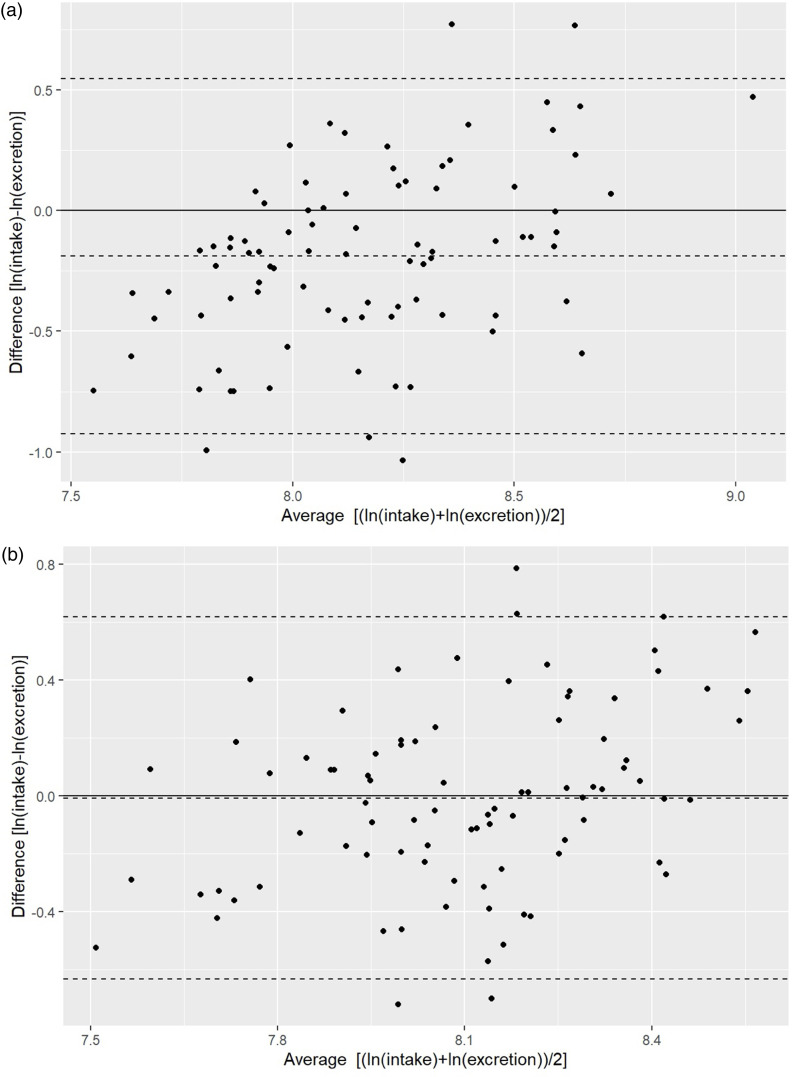
**a.** Bland–Altman plots of log dietary sodium intake (mean of 2 d) and log predicted sodium intake, as estimated by the calibrated Toft predictive equation. The horizontal dashed line indicates the mean of the differences. The upper and lower dotted lines represent the upper and lower 95 % CI of agreement, which should comprise 95 % of the values in the range of the 2-fold SD (d ± 1·96 × SD) of the mean differences. **b.** Bland–Altman plots of log dietary potassium intake (mean of 2 d) and log predicted potassium intake, as estimated by the calibrated Kawasaki predictive equation. The horizontal dashed line indicates the mean of the differences. The upper and lower dotted lines represent the upper and lower 95 % CI of agreement, which should comprise 95 % of the values in the range of the 2-fold SD (d ± 1·96 × SD) of the mean differences.

The Bland–Altman plots for assessing bias between estimated dietary intake (two-day recall) and estimated sodium and potassium excretion (from FMV urine samples) are presented in Fig. 1 for the predictive equations with the strongest correlation (Table 4, highlighted in bold). The relative measurement error remained consistent across the entire range of mean intake-excretion levels examined in the study. For sodium, the average ratio of intake and excretion, as predicted by the Toft equation, is 0.83, denoting a systematic underestimation of intake of 17%, with the upper limit of agreement at about 1.73 and the lower limit of agreement at about 0.40 (Fig. 1a). Conversely, for potassium, the average ratio of intake and excretion, as predicted by the Kawasaki equation, is 0.99, revealing almost no bias (underestimation of 1%), with the upper limit of agreement at about 1.85 and the lower limit of agreement at about 0.53 (Fig. 1b).

## Discussion

### Can spot samples reliably estimate the 24-h urine excretion of sodium and potassium? Limitations of both spot and 24-h urine collection methods

In the current study, we assessed the validity of predictive equations of FMV urine to estimate the sodium and potassium 24-h urine excretion. The main finding of this study was that although the predictive equations were moderately to strongly correlated with the 24-h urine excretion of sodium and potassium, they present a high risk of under or overestimating the excretion levels. The predictive equations using the FMV urine samples still underperform in estimating the daily excretion of sodium and potassium.

Estimating sodium excretion by spot urine samples has been widely investigated in different populations to identify individuals below or above the WHO threshold of 5 g/day salt intake^(24)^. However, estimates from spot urine significantly differ from 24-h urine measurements according to the equation used, collection time, ethnicity, and whether the spot urine was included in the 24-h sample^(16,24)^. For the Portuguese population, we found that when using the FMV urine samples to estimate the 24-h sodium excretion, the INTERSALT (with or without K) equation showed the strongest correlation. At the same time, it tended to underestimate the sodium excretion. Nevertheless, it is important to mention that the results should be interpreted cautiously, given the p-value of 0.036 and the wide confidence interval when considering the INTERSALT (with K) equation. The tendency of the INTERSALT equation to underestimate 24-h sodium excretion has already been reported for the Portuguese population^(16,42)^. Another study found that the INTERSALT equation can underestimate sodium excretion^(21)^. The INTERSALT equation was initially determined using data from different ethnic groups of multiple countries^(16)^, which may lead to systematic errors when computing for a specific population^(43)^. Conversely to the INTERSALT method, we found that the Kawasaki equation, although with a moderate correlation, considerably overestimated the 24-h urine excretion of sodium. One potential reason contributing to the comparatively lower performance of the Kawasaki equation in our study could be its original development for the Japanese population^(14)^, and the population-based adjustments likely need to be revised for Western populations. Other studies have also reported a systematic tendency of the Kawasaki equation to overestimate the mean sodium population excretion^(21,24)^. A systematic review, including 29 studies and 10 414 participants from 34 countries, identified the Kawasaki equation from spot urine samples to display the poorest performance in estimating the 24-h sodium excretion^(24)^. Considering all the six equations investigated, we found that the most predictive and reliable equation for the Portuguese population was the Toft equation, displaying a moderate correlation with the 24-h measurements and without risk of over or underestimating the sodium excretion. The Toft equation was initially developed and tested in the Danish population^(18)^ and thus more adjusted to the European population, which may explain its higher performance in the Portuguese population. Similarly to other reports^(20–24)^, 24-h sodium excretion predicted by equations using FMV urine values does not correlate strongly with the measured 24-h sodium excretion.

Estimating potassium excretion by spot urine samples has received less focus in published research. We found that for the Portuguese population, both the Tanaka and Kawasaki equations using FMV urinary potassium levels showed a moderate correlation and a substantial risk of underestimating the 24-h urine excretion of potassium. A large populational study collecting FMV urine samples from 1083 individuals from 11 countries found that the Kawasaki performed better than the Tanaka equation to estimate the 24-h potassium excretion^(26)^. Similarly, another sizeable populational study, the only one developed in Portugal to date, which assessed the validity of the estimation of 24-h urinary sodium and potassium excretion obtained through four formulae using occasional urine samples, also reported better performance using the Kawasaki equation. Still, both equations underestimate the 24-h potassium excretion^(42)^. Several other studies have highlighted the poor performance of Tanaka and Kawasaki predictive equations in estimating potassium excretion^(21,44)^. The poor performance of Tanaka^(15)^ and Kawasaki equations^(14)^ may again be related to their creation and testing in the Japanese population. Thus, they are less adequate for use in the Western population.

When predictive equations from FMV urine samples were calibrated as mentioned above, their performance was generally improved, except for the Mage equation. The level of agreement with the measured 24-h sodium excretion increased from fair to moderate with the calibration of Kawasaki and Toft equations. Regarding the potassium excretion, the level of agreement increased from slight to fair when the Tanaka equation was calibrated.

The overall weak to moderate correlations found between predictive equations and measured 24-h urine samples indicate the limitations of the FMV urine samples in estimating the daily sodium and potassium excretion. Predictive equations were often created based on specific populations that reflect ethnic groups and patterns of sodium and potassium intake over the day, which will downplay their applicability and reliability when used with other populations. In fact, the timing of spot collection has been widely debated as an influencing factor for predicting performance^(12,25,44,45)^. We collected FMV urine samples to standardise the collection time across our entire sample. Consequently, they are limited to the characteristics of morning urine and do not reflect the variations in the 24-h urinary sodium and potassium excretion levels throughout the day according to the circadian rhythm, hydration level and sodium intake. These confounding factors can influence excretion levels, which are higher in the afternoon and evening than at night or in the morning^(31)^, resulting in within-day variation^(25)^. However, there is conflicting evidence on the most suitable time to collect the spot sample. While some studies suggest that morning samples display the best performance^(20,46)^ (with the second-morning void being more reliable than the first^(47)^), other studies report that afternoon and evening urine spot samples perform better^(14,17,31,48)^, and other studies have found no significant differences^(45)^.

Further clarification on the optimal collection time is warranted to inform on how to improve the sodium and potassium intake estimate using equation-based methods from spot urine samples. The number of spot collections has also been linked to the 24-h estimation accuracy. Some authors advocate collecting three spot urine samples to improve accuracy and reduce the effect of within-person variation within urinary excretion^(49)^. In contrast, others have found that six daytime spot samples might replace a 2-day 24-h urinary excretion^(50)^. Although collecting multiple urine spots can be burdensome^(10,13)^, they are more feasible than multiple 24-h collections and can be implemented to calibrate the bias of casual urine spots^(43)^.

Despite the limitations of using spot urine samples, we should continue our efforts to improve the currently available methods to estimate the sodium and potassium intake from spot samples. Spot urine collection uses a small sample of urine from a single void. This method yields many advantages for population monitoring as it can be easily incorporated into broader population health and/or nutrition surveys because samples can be collected in a single encounter, easily stored, and without the potential for under- or overcollection. Although the 24-h collection should remain the ‘gold standard’ until other more straightforward methods prove reliable and accurate, it is still a cumbersome procedure with often poor compliance, which can be affected by several factors, including age, gender, socioeconomic status and standard of the collection process^(51)^. The high burden and difficulty in collecting complete 24-h urine samples highlight the need for less resource-intensive and more practical methods. Finding a simple and reliable method is a cornerstone to establishing reliable benchmark levels of dietary sodium and potassium intake for policymakers who make decisions and devise strategies to control salt intake and implement routine monitoring programmes of changes over time among various target groups.

### How do spot urine samples correlate with dietary recalls?

Due to its practicality, we wanted to explore further how spot urine samples correlate with dietary recalls. Our previous study evaluated the accuracy of the new software eAT24 used to assess dietary intake in the IAN-AF survey against urinary biomarkers: nitrogen, potassium, and sodium^(27)^. We examined differences between estimates from 24-h dietary recalls and urine measures, and correlation coefficients were calculated.

Therefore, our secondary objective was to assess the correlation between estimated sodium and potassium intake from dietary recalls and the urinary excretion predicted by calibrated equations using FMV urine values. This enables us to understand if spot urine samples can be a feasible alternative to the more resourceful 24-h urine collection.

We found that one-day or mean of two-day dietary recalls for sodium and potassium were, at best, moderately correlated with the calibrated equation-based estimations from FMV urine. Higher correlations were observed when the mean of two-day dietary recalls for sodium and potassium was compared with the predicted urine levels. However, our previous study^(27)^ found that when considering the mean intake calculated using the two days of recall, the correlation coefficient between dietary recalls and 24h-urine samples improved for potassium but not sodium.

The two-day dietary recall of sodium intake showed a higher correlation with the calibrated Toft equation, although the intake was systematically underestimated. The correlation between two-day dietary recall of sodium intake and 24-h urinary sodium excretion was 0.21^(27)^. The correlation increased to 0.45 when the urinary sodium excretion was estimated through the Toft calibrated predictive equation. This unexpected increase highlights the significance of the supplementary variables included in the equation, sex, age, height, and weight (in addition to spot sodium and spot creatinine), in providing a more comprehensive understanding and explanatory power regarding the variability in sodium intake. Interestingly, this was not observed for potassium estimations, in which a correlation coefficient decrease was observed from 0.53 to 0.35.

The results from the Bland-Altman plots suggest no substantial differences between methods for assessing potassium intake, as dietary recalls underestimate potassium intake by only 1% compared to FMV (spot) urine, as estimated by the calibrated Kawasaki predictive equation. These results are similar to our previous study, in which dietary potassium was overestimated by only about 4% relative to 24-h urine. Interestingly, the limits of agreement for dietary potassium vs. FMV urine (0.53 to 1.85) are slightly narrower than those for dietary potassium vs. 24-h urine (0.52 to 2.09)^(27)^, suggesting lower individual variability with the FMV-based approach. For sodium, we found that dietary sodium substantially underestimates FMV urine by 17%, as estimated by the calibrated Toft predictive equation, which is higher than the 13% underestimation observed when comparing dietary sodium with 24-h urine^(27)^. Notably, the limits of agreement for dietary sodium and FMV urine (0.40 to 1.73) are narrower than those for 24-h urine (0.31 to 2.49)^(27)^, indicating lower individual variability with the spot-based method.

Overall, these findings highlight that spot urine samples, particularly when using calibrated predictive equations, offer a practical and moderately accurate alternative to 24-h urine collection for estimating population-level sodium and potassium intake. While dietary recalls for potassium show reasonable alignment with urinary biomarkers, the inclusion of supplementary variables in predictive equations significantly enhances the correlation for sodium estimates, underscoring the value of these models in addressing the inherent variability of sodium intake. The narrower limits of agreement for FMV urine compared to 24-h urine suggest that spot urine methods provide more consistent estimates, making them a feasible and resource-efficient choice for large-scale nutritional epidemiology studies. However, nutrient-specific considerations are crucial, as sodium and potassium exhibit differing levels of correlation and variability, necessitating tailored approaches to optimise accuracy and practicality.

### Limitations of the current study

Our study is not exempt from some limitations. Our sub-sample did not recruit participants from all geographic regions of the IAN-AF sample, and we thus advise some caution when extrapolating the results of our study for the entire Portuguese population. However, we expect a similar performance for the remaining Portuguese general population. The small sample size (n=86) is another limitation of our study, especially when considering populational averages. Still, we believe that can be representative enough for the data analyses performed in our study. Notwithstanding, such a small sample size precluded us from completing subgroup analyses to explore the consistency of our findings across subgroups defined by age, sex, ethnicity, and other baseline characteristics.

Concerning the validity of our urine samples, we did not use any criteria to evaluate the appropriateness of FMV urine samples, for example, urine creatinine concentration or specific gravity, which are recognised markers of several kidney dysfunctions^(49)^. However, we included healthy individuals, not diuretic users, to ensure that none of the participants had kidney diseases. As referred to in the previous study, we did not validate the completeness of 24-h urine samples using para-aminobenzoic acid (PABA) but instead implemented other widely used criteria^(27)^. Even so, using different indirect methods to assess the completeness of urine collections can lead to important changes in dietary intake/excretion^(52)^ estimates and result in under- or overestimating intake^(6)^.

At the time we conducted this study, we used a 0.86 (for sodium) and 0.80 (for potassium) correction factor to reflect the extra-renal losses^(8)^. This correction was based on contemporary knowledge that 24-h urine collections capture between 86% to 93% of average sodium intake^(8,9)^, with the remaining excreted via sweat, intestinal fluids and saliva^(19)^. As shown by the previous studies, sodium excretion rate varies among individuals; thus, the choice of correction factors may impact the results of predictive equations^(8,9)^.

The spot urine samples collected in our study (FMV samples) were not independent of the 24-h urine collections, which may have overestimated the strength of the agreement. Moreover, collecting only FMV spots precluded us from analysing the effect of the timing of urine collection. The WHO’s STEPwise approach to Surveillance protocol includes spot urine collection to estimate the salt intake. Still, it does not recommend the timing of the urine collection or which equation should be used^(53)^.

### Conclusion

This study demonstrates that predictive equations to estimate sodium and potassium excretion from FMV urine samples do not sufficiently correlate with 24-h urine collections, which remain the gold standard. Despite moderate to strong correlations for some equations, sodium and potassium estimates consistently show systematic biases, either overestimating or underestimating excretion levels.

Nevertheless, FMV urine samples used with calibrated predictive equations offer a practical and moderately accurate alternative for population-level sodium and potassium intake assessments. Incorporating additional variables such as sex, age, height, and weight improves the accuracy of these equations, especially for sodium. Moreover, the narrower limits of agreement observed for FMV urine compared to 24-h collections suggest it provides relatively consistent estimates for population-level studies, making FMV urine a feasible and resource-efficient option for large-scale nutritional epidemiology studies when 24-h urine collection is impractical.

To support public health policies and health promotion programmes, it is essential to establish reliable benchmark levels for spot urinary sodium and potassium. These benchmarks would enable the evaluation of dietary trends across diverse population groups and inform effective intervention strategies.

Future research should focus on nutrient-specific approaches, refining predictive equations, optimising the timing of spot urine collection, and evaluating methods to reduce systematic biases. Such advancements could enhance the accuracy and applicability of FMV urine samples, bridging the gap between practicality and precision in dietary sodium and potassium assessments.

## Supporting information

Goios et al. supplementary material 1 Goios et al. supplementary material

Goios et al. supplementary material 2 Goios et al. supplementary material

## Supplementary material

The supplementary material for this article can be found at https://doi.org/10.1017/jns.2025.16
