# Supplementary material for: Estimating sodium and potassium intakes in a Portuguese adult population: can first-morning void urine replace 24-hour urine samples?
Source: J Nutr Sci. 2025 Mar 26;14:e29. doi: 10.1017/jns.2025.16 (PMC11955307; doi:10.1017/jns.2025.16)
Supplement: Goios et al. supplementary material 1 — Goios et al. supplementary material [file S2048679025000163sup001.docx]

**Supplementary** **Table 1 -** Demographic, anthropometric characteristics, and energy intake of the analytic sample by sex (n=86)

|  | **Women (n=43)** | | **Men (n=43)** | | **Total (n=86)** | |
| --- | --- | --- | --- | --- | --- | --- |
|  | **Mean** | **SD** | **Mean** | **SD** | **Mean** | **SD** |
| Age, *years* | 46.2 | 14.9 | 50.3 | 15.3 | 48.3 | 15.2 |
| Weight, *kg* | 67.1 | 11.6 | 80.5 | 11.5 | 73.8 | 13.3 |
| Energy intake [one day of recall], *kcal* | 1674 | 521 | 2513 | 989 | 2094 | 892 |
| Energy intake [mean of two days of recall], *kcal* | 1682 | 464 | 2441 | 858 | 2062 | 785 |
| BMI category^a,^ *n (%)* |  |  |  |  |  |  |
| Normal weight | 17 (39.5) | | 12 (27.9) | | 29 (33.7) | |
| Overweight | 19 (44.2) | | 25 (58.1) | | 44 (51.2) | |
| Obesity | 7 (16.3) | | 6 (14.0) | | 13 (15.1) | |

**Legend:** SD, Standard Deviation; BMI: Body Mass Index.

^a^ BMI category (kg/m^2^): normal weight (18.5 to <25.0), overweight (25.0 to <30.0), obesity ($\geq$30.0)
