# Supplementary material for: Estimating sodium and potassium intakes in a Portuguese adult population: can first-morning void urine replace 24-hour urine samples?
Source: J Nutr Sci. 2025 Mar 26;14:e29. doi: 10.1017/jns.2025.16 (PMC11955307; doi:10.1017/jns.2025.16)
Supplement: Goios et al. supplementary material 2 — Goios et al. supplementary material [file S2048679025000163sup002.docx]

**Supplementary** **Table 2 - Predictive equations used to estimate 24-h urine excretion of sodium from spot urine samples**

| **Method** | **Published time** | **Study population** | **Age range (years)** | **Urine specimen** | **Predictive formula (mmol / 24-h)** |
| --- | --- | --- | --- | --- | --- |
| Kawasaki ^(1, 2)^ | 1993 | Japanese men (n=87) and women  (n=81) | 20-79 | Second morning voiding urine | Male: 16.3 × {spot Na (mmol/l)/ [spot Cr (mg/dL) x 10] × [ − 12.63 × age (years) + 15.12 × weight (kg) +7.39 × height (cm) – 79.9]}^0.5^  Female: 16.3 × {spot Na (mmol/l)/[spot Cr (mg/dL) x 10] × [ − 4.72 × age (years) + 8.58 × weight (kg) + 5.09 × height (cm) − 74.5]}^0.5^ |
| Tanaka ^(3)^ | 2002 | Japanese men (n=295) and women (n=296) | 20-59 | Casual spot urine | 21.98 × {spot Na (mmol/l)/[spot Cr (mg/dL) × 10] ×[− 2.04 × age (years) + 14.89 × weight (kg) + 16.14 × height (cm) − 2244.45]}^0.392^ |
| Mage ^(4)^ | 2008 | Used to estimate urine pesticide and  chemical exposure  with NHANES urine  specimens in men (n=249) and women (n=18) | 18-92 | Casual spot urine | Male: [spot Na (mmol/L)/(spot Cr (mg/dL) × 10)] × {0.00179 × [140 − age (years)] × [weight (kg)^1.5^ × height (cm)^0.5^] × [1 + 0.18 × A × ]}, where A is African American or black race = 1, other race = 0  Female: [spot Na (mmol/L)/(spot Cr (mg/dL) × 10)] × {0.00163 × [140 − age (years)] × [weight (kg)^1.5^ × height (cm)^0.5^] × [1 + 0.18 × A]}, where A is African American or black race = 1, other race = 0 |
| INTERSALT without Potassium ^(5)^ | 2013 | Western (North American and European) men (n=2841) and women (n=2852) | 20-59 | Casual spot urine | Male: {23.51 + [0.45 × spot Na (mmol/L)] – [3.09 × spot Cr (mmol/L)] + [4.16 × BMI (kg/m^2^)] + [0.22 × age (years)]}  Female: {3.74 + [0.33 × spot Na (mmol/L)] – [2.44 × spot Cr (mmol/L)] + [2.42 × BMI (kg/m^2^)] + [2.34 × age (years) − [0.03 × age^2^(years)]} |
| INTERSALT with Potassium ^(5)^ | 2013 | Western (North American and European) men (n=2841) and women (n=2852) | 20-59 | Casual spot urine | Male: {25.46 + [0.46 × spot Na (mmol/L)] – [2.75 × spot Cr (mmol/L)] − [0.13 × spot K (mmol/L)] + [4.10 × BMI (kg/m^2^)] + [0.26 × age (years)]}  Female: {5.07 + [0.34 × spot Na (mmol/L)] − [2.16 × spot Cr (mmol/L)] − [0.09 × spot K (mmol/L)] + [2.39 × BMI (kg/m^2^)] + [2.35 × age (years)] – [0.03 × age^2^ (years)]} |
| Toft ^(6)^ | 2014 | Danish men (n=102) and women  (n=371) | 18-65 | Casual spot urine | Male: 33.56 × {spot Na (mmol/L)/(spot Cr (mg/dL) × 10) × [− 7.54 × age (years) + 14.15 × weight (kg) + 3.48 height (cm) + 423.15]}^0.345^  Female: 52.65 × {spot Na (mmol/L)/(spot Cr (mg/dL) × 10) × [−6.13 × age (years) + 9.97 × weight (kg) + 2.45 × height (cm)+342.73]}^0.196^ |

**References**

1. Kawasaki T, Itoh K, Uezono K *et al.* (1993) A simple method for estimating 24 h urinary sodium and potassium excretion from second morning voiding urine specimen in adults. *Clin Exp Pharmacol Physiol* 20, 7-14.

2. Kawasaki T, Uezono K, Itoh K *et al.* (1991) [Prediction of 24-hour urinary creatinine excretion from age, body weight and height of an individual and its application]. *Nihon Koshu Eisei Zasshi* 38, 567-574.

3. Tanaka T, Okamura T, Miura K *et al.* (2002) A simple method to estimate populational 24-h urinary sodium and potassium excretion using a casual urine specimen. *J Hum Hypertens* 16, 97-103.

4. Mage DT, Allen RH Kodali A (2008) Creatinine corrections for estimating children's and adult's pesticide intake doses in equilibrium with urinary pesticide and creatinine concentrations. *J Expo Sci Environ Epidemiol* 18, 360-368.

5. Brown IJ, Dyer AR, Chan Q *et al.* (2013) Estimating 24-hour urinary sodium excretion from casual urinary sodium concentrations in Western populations: the INTERSALT study. *Am J Epidemiol* 177, 1180-1192.

6. Toft U, Cerqueira C, Andreasen AH *et al.* (2014) Estimating salt intake in a Caucasian population: can spot urine substitute 24-hour urine samples? *Eur J Prev Cardiol* 21, 1300-1307.
